# Hepatitis in a Dog

**Published:** 1883-10

**Authors:** Mark L. Frey

**Affiliations:** New York City


### III HEPATITIS IN A DOG.

BY MARK L. FREY, D.V.S.

On the 8th of June I was called to see a dog—the history of the case being as follows:

The owner noticed that the animal appeared dull and restless, that he had a high fever, continually panting and that he had vomited twice during the day.

Upon careful examination I found the above symptoms to be correct with also a slight tenderness in the right side of the abdomen and bowels constipated.

I at first mistook it for enteritis, but after carefully watching the patient I noticed that the symptoms, though not very unlike, were less severe and with less prostration of strength.

I was now at a loss as to the nature of the trouble and asked the owner to note all the symptoms he noticed, and that I would call the next day. I, however, prescribed

℞

Pil. Hydrarg, et,

Aloes Barb.. aa, ʒii

Fiat massa in pil vi dividend.

Equibus donantur, binæ nocte.

And also recommended that the animal be bathed in warm water two or three times a day.

The following morning the symptoms of the previous day were more severe and prominent, the urine was of a deep yellow color, the skin appeared to be likewise universally tinged, but the coverings of the eyes and mouth more particularly so. I then arrived at the conclusion that the dog was suffering from Hepatitis.

The pills were continued until that night, when I considered the bowels well open; after which I prescribed the following, to be given every four hours:

℞

Pulv. Digitalis, gr. viii.
Pulv. Antimonialis gr. xvi.
Potassæ Nitrat, P. -) i
Misce.

And divide into twelve powders. A blister was also applied to the right side of the abdomen.

This treatment was carefully carried out and on the fifth day the animal was discharged cured.

NEW YORK CITY.

